# Novel Strategies in Artificial Organ Development: What Is the Future of Medicine?

**DOI:** 10.3390/mi11070646

**Published:** 2020-06-30

**Authors:** Marta Klak, Tomasz Bryniarski, Patrycja Kowalska, Magdalena Gomolka, Grzegorz Tymicki, Katarzyna Kosowska, Piotr Cywoniuk, Tomasz Dobrzanski, Pawel Turowski, Michal Wszola

**Affiliations:** Foundation of Research and Science Development, 01-793 Warsaw, Poland; marta.klak@fundacjabirn.pl (M.K.); tomasz.bryniarski@fundacjabirn.pl (T.B.); patrycja.kowalska@fundacjabirn.pl (P.K.); magdalena.gomolka@fundacjabirn.pl (M.G.); grzegorz.tymicki@fundacjabirn.pl (G.T.); katarzyna.kosowska@fundacjabirn.pl (K.K.); piotr.cywoniuk@fundacjabirn.pl (P.C.); tomasz.dobrzanski@fundacjabirn.pl (T.D.); pawel.turowski@fundacjabirn.pl (P.T.)

**Keywords:** 3D bioprinting, organ-on-a-chip, bionic tissue, bioink, cell culture

## Abstract

The technology of tissue engineering is a rapidly evolving interdisciplinary field of science that elevates cell-based research from 2D cultures through organoids to whole bionic organs. 3D bioprinting and organ-on-a-chip approaches through generation of three-dimensional cultures at different scales, applied separately or combined, are widely used in basic studies, drug screening and regenerative medicine. They enable analyses of tissue-like conditions that yield much more reliable results than monolayer cell cultures. Annually, millions of animals worldwide are used for preclinical research. Therefore, the rapid assessment of drug efficacy and toxicity in the early stages of preclinical testing can significantly reduce the number of animals, bringing great ethical and financial benefits. In this review, we describe 3D bioprinting techniques and first examples of printed bionic organs. We also present the possibilities of microfluidic systems, based on the latest reports. We demonstrate the pros and cons of both technologies and indicate their use in the future of medicine.

## 1. Introduction

Currently, animal testing is the most popular technique for examining an organism’s response to a biologically active compound. Among various animals used for this purpose, we can distinguish rats, mice, rabbits, monkeys, and pigs [1]. Owing to the development of biotechnological techniques, for several years now, there has been a trend of minimalizing the number of used animals.

**Figure 1:**
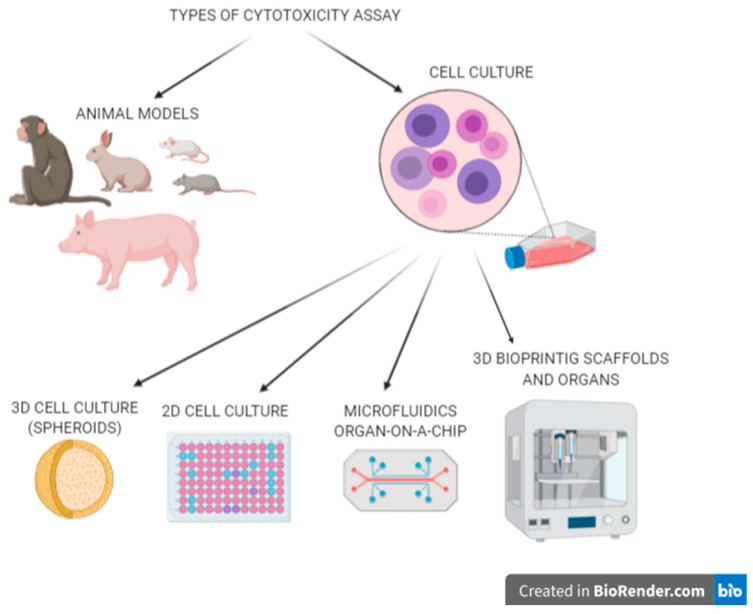
Types of biological materials and techniques used in cytotoxicity assays during potential new drug testing.

Another big issue with reliable biological models in laboratories is the common use of 2D cell culture, which are far from perfect [2]. Results obtained from tests on 3D cultures are proved to be much more dependable [3]. They are mainly based on the co-cultivation of various cell types. Furthermore, promising alternatives arise with the generation of a microfluidic system called organ on-a-chip (OOC) [4]. Moreover, the latest achievements present the possibility of 3D bioprinting of entire organs to study their response. Laminar fluid flow in microfluidic systems and the three-dimensional space obtained in the bioprinting process are key factors that enable the coexistence and observation of different types of interacting cells. In the graph below (Figure 1), we present types of techniques used to examine the cytotoxicity of potential drugs [3]. The objective of this article is to systematize existing knowledge considering the production of functional tissue models and their significant role in biomedical engineering.

## 2. From 2D to 3D Culture

The monolayer method of cell culture has been used for many years as a tool for various types of drug screenings, toxicity assessment and studying cells’ molecular mechanisms [5]. There are many advantages of 2D cultures; above all, we value them for their simplicity, ease of technical manipulation, and scalability. On the other hand, the monolayer method of cell culture has many disadvantages, e.g., a lack of dimensionality of natural tissue structures or tumor structures and a lack of elements of the microenvironment such as an ECM (extracellular matrix). The isolation of tissue from the native environment and placing it in 2D culture is associated with changes in cell physiology, in the way they are divided and in response to various external stimuli [6]. Moreover, a tendency to change gene expression, splicing, topology, as well as cell biochemistry also has been shown [7].

**Figure 2:**
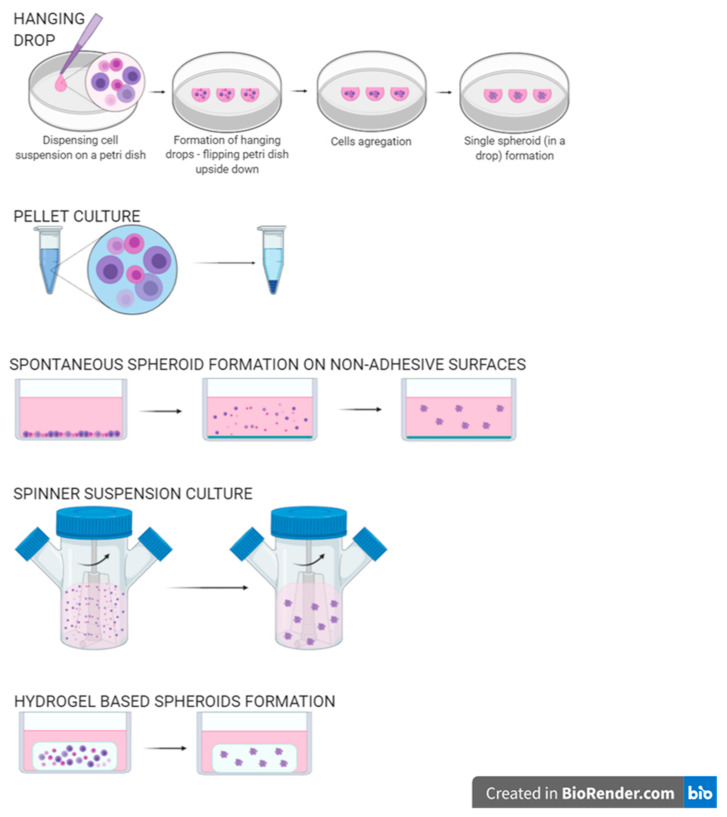
Methods of cell spheroids creation.

Numerous limitations of 2D cell cultures initiated work on three-dimensional (3D) cultures. There are several methods to create spheroids, including single cell-type 3D culture or even a 3D co-culture of various cells that imitate native tissue (Figure 2). A 3D cell culture mimics reality far better and it helps thoroughly examine how cells and tissues forming entire organs are shaped and also examine the interactions between them together with the influence of mechanical stimuli [8]. The beginning of three-dimensional cell cultures dates back to the 1970s. One of the first such cultures was agar-cultivated and produced by Hamburger and Salmon [9]. A lot of time has passed since, and the development of proper biomaterials and techniques such as bioprinting and microflow revolutionized all fields within this subject. 

However, sophisticated techniques for creating 3D scaffolds from a wide range of biomaterials and hydrogels based on ECM are not sufficient to restore functional organs. Key problems limiting the prospects for the use of such models as a substitute for animal studies are, among others, maintaining an adequate permeability of chemical compounds, oxygen, and obtaining a mechanically active environment. There is also a serious problem in the effective detection of tissue physiology, mainly in the ion diffusion gradient and the exchange of compounds, e.g., between epithelium and vascular endothelium [8].

Undoubtedly, in order to effectively use 2D and 3D cultures as a platform for research on the functioning of organs and tissues, innovative engineering solutions are required. The combination of microplating (the microchip industry) together with 3D bioprinting has become an excellent solution. Systems based on microflow have enabled rapid molecular gradient changes (step gradient), low Reynolds numbers, and a complete laminar flow of fluids in the channels [10]. The benefits of microfluidics led to its application in both 2D and 3D culture systems.

## 3. Organs on-a-Chip 

**Figure 3:**
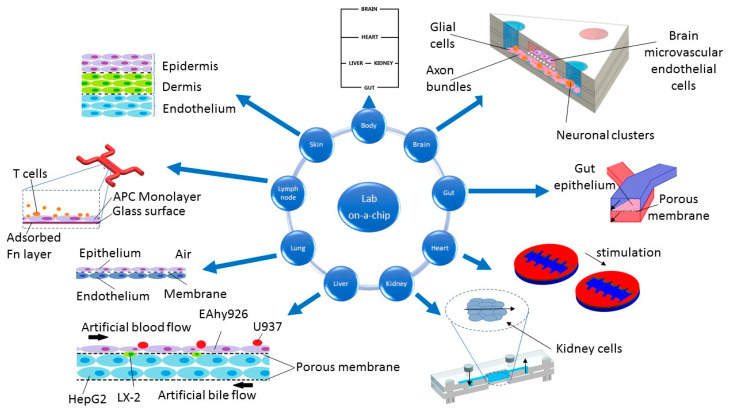
Example uses of lab on-a-chip systems. All figures within this figure were prepared on the basis of original papers (body [15], brain [16], gut [17], heart [18], kidney [19], liver [20], lung [21], lymph node [22], and skin [23]).

Created in 1990, a landmark article by Manz et al. [11] introduced the field of miniaturized comprehensive chemical analysis system (µTAS). This system aims to carry out reactions in very small volumes but reflecting onto a larger scale. It has been successfully used in other fields such as biology, tissue engineering, chemistry, and physics, and the system itself has been referred to as “microflow” [12]. It thoroughly controls fluids through microchannels, with volumes ranging from 10^−9^ to 10^−18^ liters and lengths up to several hundred micrometers. Microfluidic systems used in laboratory experiments—“Lab on-a-chip”—were the beginning of the currently developing “organ-on-a-chip” technology, which significantly influences medical sciences. In OOC systems, a cell culture takes place in chambers and channels that are designed to mimic the biology and physiology of tissues or complete organs [13]. Restoring their function as realistic models requires maintaining appropriate conditions such as pressure, pH, flow rate, nutrient content, and the presence of toxins or drugs. To summarize, OOC technology allows conducting in vitro tests of organs, tissues or 3D cultures in controlled conditions. To date, in vitro studies have been performed on cardiovascular, respiratory, nervous, digestive, endocrine and skin-like systems (Figure 3) [14].

### 3.1. Liver-on-a-Chip

The high metabolic activity of the liver makes it a crucial organ for maintaining health. The homeostatic processes in this organ are supported by numerous cells that form an integral unit. Hepatocytes, sinusoidal endothelial cells, Kupffer cells and stellate cells communicate through direct contact and by diffusion. Even though this organ has a highly regenerative nature, the liver is immensely exposed to damage caused by chronic diseases and viral infections [24]. The main problem of standard 2D hepatocyte cultures is to sustain high functionality for a long time. Modern perfusion systems enable the flow of nutrients so that faster hepatocyte detoxification is possible, resulting in better functionality and the experiment extension. 

Non-alcoholic fatty liver disease (NAFLD) is the most common chronic disease of this organ and is a major cause of liver failure. To date, studies on the pathogenesis of NAFLD have mainly focused on hepatocytes alone that may not be able to map the complex liver microenvironment. Lasli et al. [25] presented an in vitro culture system of human liver cancer cells (HepG2) with human umbilical vein endothelial cells (HUVEC) as chip spheroids. This experiment allowed monitoring of the functionality of spheroids due to an increased secretion of albumin with HepG2–HUVEC interactions. The effectiveness of the anti-static drug was demonstrated, which caused the intracellular lipid levels to return to baseline faster. On the other hand, Deng et al. [26] developed a demountable device on a chip to investigate the pathophysiology of single non-parenchymal cell processes in alcoholic liver disease (ALD). The device was capable of recreating the alcohol-induced damage process of liver parenchymal cell lines. It also helped to understand the interactions between different types of liver cells during the course of ALD.

### 3.2. Kidney-on-a-Chip

The kidneys are one of the most difficult organs to mirror due to the variety of tissues with different functions. This organ consists of glomerular cells, proximal and distal tubules, Henle cell loops, thick ascending limb cells, interstitial kidney cells and kidney endothelial cells. The main functions of the kidney are fluid level control and toxin elimination thanks to filtration and reabsorption mechanisms [27]. Thus, it is extremely important for the kidney-on-a-chip biomimetic system to preserve interactions between all kidney-type cells. It is also important to maintain adequate osmotic and electrochemical pressure. The OOC models developed so far are able to reflect the physiology of the glomeruli and proximal and distal tubules. However, the reconstruction of all kidney components has not yet been achieved [28].

Tian et al. [29] created a hepatorenal system on a chip based on precisely cut tissue slices. This study used tissue because of much better chemokine reconstruction of the microenvironment than standard OOC using individual cell lines. Thanks to the biomimetic microsystem, it was possible to model the organotropism of extracellular follicles in breast cancer. Extracellular follicles of breast cancer have been shown to exhibit strong liver tropism instead of kidney tropism, according to both the flow model and animal models [29]. One of the last contributions to this field was made by Theobald et al. [30], who demonstrated that the use of a microfluidic platform mimics in vitro D3 vitamin metabolism. In this system, two chambers imitating kidneys and a liver were used, and into those chambers, the bioactive form of D3 vitamin was introduced [30]. Analysis of metabolic products confirmed their anti-cancer activity in acute leukemia cells. A study performed by the authors suggests that microfluidic systems of this type can be successfully used to mimic the in vivo metabolism of various microelements and xenobiotics.

Most microfluidic studies focus on drug toxicity assessment using renal proximal tubular epithelial cells. However, in vitro models are difficult to obtain. Yin et al. [31] developed a device consisting of a three-layer flow chip. Renal proximal tubular epithelial cells (RPTEC) and perithelial capillary endothelial cells (PCEC) were used in this experiment to assess cisplatin-induced nephrotoxicity. The protective effect of cimetidine has been demonstrated [31]. Furthermore, an interesting tool for screening multi-drug toxicity was designed by Lin et al. [32]—a microfluidic model using hepatic spheroids and proximal renal tubules. This study showed the effect of ciclosporin A (CsA) on the course of cytotoxicity and a decrease in the concentration and cytotoxicity of CsA by rifampicin.

### 3.3. Gut-on-a-Chip

In vitro models are not capable of faithfully reproducing the intestinal microenvironment, mainly due to the complexity of mechanical phenomena such as fluid flow and cyclic peristaltic movement. Another major limitation of in vitro models is the maintenance of live microbial cultures on the surface of the X-rayed intestinal epithelium because of the inability to maintain the culture for a long time. This is a serious problem because the microbiome plays a significant role in functioning of the intestinal barrier, metabolism, drug absorption, and in course of many types of inflammatory bowel disease (IBD) [33]. To overcome these limitations, microfluidic intestinal systems have been developed with capabilities far exceeding those offered by conventional methods of cell culture. In one recent study [34], an intestine was created on a chip, and it used an independent modulation of parameters such as peristaltic movement and the presence of immune cells or microbes. This study proved that the lack of a mechanism enabling epithelium deformation is sufficient to cause bacterial overgrowth of the small intestine, similar to that observed in some patients with obstruction of the small intestine. More microfluidic studies related to the maintenance of the microbiome can be found in the review by Bein et al. [35].

Therapeutic, as well as accidental γ radiation can give rise to serious intestinal damage, i.e., the shortening of intestinal villi, increased microvascular endothelial apoptosis, mucosal barrier damage, and many others. In one study [36], a microfluidic device lined with intestinal epithelial cells and vascular endothelial cells was created. This research was aimed at modeling radiation damage and assessing the effectiveness of radiation-protective drugs in vitro. The results of this work are consistent with animal models. Dimethyloxalylglycine has also been proved to be a potential preventive radioprotective drug. The above studies suggest that microfluidic models of the intestinal system can be successfully used to track the course of diseases of various origins, as well as test new drugs.

### 3.4. Lung-on-a-Chip

The key function of the respiratory system is to supply oxygen, remove carbon dioxide, maintain blood pH and filter xenobiotics. The precise structure of tissues and a number of complex biochemical and biophysical factors allow gas–liquid exchange, since the membrane is only a few microns thick [37]. Conventional culture methods do not provide cells with mechanical stimuli, such as shear stress, stress or compression, or physical stimuli such as substrate rigidity and specific dimensional and geometric nanostructures. The above factors play an important role as physiological indicators of proliferation, contractility, cell motility and tissue morphogenesis. Microflow models are able to accurately map natural conditions; therefore, they show great potential in studying the physiology and etiology of human lung diseases [38]. Therefore, it seems understandable that in vitro lung and respiratory membrane modeling is a huge challenge for modern tissue engineering. 

One of pioneering studies is the biomimetic microsystem mapping the vesicular–capillary interface of the human lung described by Huh et al. [21]. This system was based on a microfluidic system with a centrally located, porous, and flexible 10 μm thick polydimethylsiloxane membrane. The device was capable of visualizing the response to bacteria and the course of inflammatory processes. The impact of mechanical deformations on toxic and inflammatory lung responses to silica nanoparticles has been demonstrated, as well as an increase in their absorption through the endothelium–epithelium interface. The results of these studies coincide with studies in mice, where a similar effect of physiological respiration on the absorption of nanoparticles has been observed. In another research, conducted by Felder et al. [39], they assessed the impact of the mechanical load. In this case, the impact of the load on wound healing of the alveolar epithelium was examined on the breathing lung-on-a-chip. As a result of the semi-open lung structure, it was possible to manually cause mechanical damage and observe the healing process during cyclic stretching. It was shown that stretching with a linear stress value of 10% and frequency of 0.2 Hz significantly disturbed the regeneration of the epithelial wound compared to static cultures. The positive effect of the rhHGF (Recombinant human Hepatocyte Growth Factor) growth factor has also been confirmed. To study the toxicity of doxorubicin (DOX) and docetaxel, the team of Asad Ullah Khalid et al. [40] developed a multi-sensor platform for lung cancer on chip, using NCI H1437 cell lines. Thanks to the applied electrical impedance through the epithelium (TEER), it was possible to monitor the toxicity of drugs in real time and the pH of the culture medium. Cell viability has been shown to decrease with increasing doxorubicin levels. According to the authors, this platform can be successfully used to track toxicity for any cancer models and organ tissues.

### 3.5. Heart-on-a-Chip

The heart is an important organ that maintains homeostasis in the body. The supply of nutrients to organs is mainly managed by specialized cells such as cardiomyocytes, which are responsible for heart contractions [41]. Myocardial infarction, hypertrophy, and atherosclerosis are some of the major diseases with high mortality around the world. Therefore, the development of studies on this organ is essential. The main obstacles for the effective study of the cardiovascular system in vitro by traditional methods result from the difficulty of mapping the native microenvironment and measuring contractility and electrophysiology. Current heart-on-a-chip models are focused primarily on the mapping of orientation and synchronous beating of cardiomyocytes in vitro. 

For effective measurement and calculation of myocardial systolic and diastolic stress, Agarwal et al. [42] proposed a heart-on-a-chip model. That model was using a semi-automatic technique consisting of thin layers of soft elastomer support less than a millimeter thick (MTF). The deflection of MTF brackets during shrinkage allowed calculating the precise stress. The same paper also presents a reusable single-channel flow micro-device for an optical test of cardiac contractility and for collecting systolic responses to drugs. In another work, Schneider et al. [43] proposed an OpenHeartWare device that allows for observation of the spatial–temporal dynamics of heart tissues with the use of bright field microscopy. In this research, the scientists examined the effect of several microenvironmental factors (such as cell density, type of hydrogels, and electrical conditioning regime) on the functional and structural assembly of stem cells. An automated and independent farming system designed by Qiao et al. [44] is also an interesting heart-on-a-chip microfluidic model. This project succeeded in the optimization of organo-like culture conditions to maintain human heart slices for 4 days. It provides medium flow, oxygenation, temperature control, static mechanical load, and electrical stimulation. According to the authors, the model is capable of long-term research on the effectiveness of drugs, gene therapies, gene editing, as well as basic research in the field of physiology of the human heart.

### 3.6. Brain-on-a-Chip

The brain is one of the most complex human organs. Even minor brain disorders or changes can have serious consequences for both mental and physical health. The functioning and genetics of the human brain are significantly different from animal models, and the architecture complexity and selectivity of the human blood–brain barrier significantly limit the use of many therapeutic drugs and antibodies. The main limitations of 2D and 3D models include no cell voltage or shear stress because of the flow. Accordingly, in vitro platforms have been developed that are capable of mimicking complex structures and physiological responses in the human brain [16]. Particularly important models are the ones based on the blood–brain barrier because of their innovative method of modeling neurodegenerative diseases and high-throughput drug screening [45]. 

Park et al. [46] described a microfluid blood–brain barrier (BBB) model that demonstrated high BBB functionality in vivo for at least one week. This system was lined with induced pluripotent endothelium, derived from human stem cells, connected to primary astrocytes and human brain pericytes. The possibility of reversible opening of BBB with hypertonic solutions enabled them to observe the interaction of the P-glycoprotein–citalopram transporter in physiological flow. According to the authors, this system is an excellent platform for researching drugs and transporters that selectively cross the blood–brain barrier, as well as for modeling CNS (central nervous system) diseases in vitro using a patient’s induced pluripotent stem cells. In another study, Koo et al. [47] present a four-chamber microfluidic system consisting of BBB with dynamic flow, membrane-free endothelial culture, and embedded extracellular matrix with neuroblastoma, microglia, and astrocytes. This rather complex BBB model aimed to study the neurotoxicity of organic phosphate-based compounds. It has been shown that phosphates are capable of crossing the blood–brain barrier and inhibiting acetylcholinesterase activity, as confirmed by in vivo studies. Undoubtedly, the articles presented in this section show the potential as a cost-effective and alternative method for research with animal models. Nevertheless, due to the extensive brain-on-a-chip theme, which is not the main topic of this work, we refer to the review of Ali Mofazzal Jahromi et al. [48], where the results of recent research are discussed in detail.

### 3.7. Skin-on-a-Chip

The skin, being the largest organ, plays a very important role in the human body. The dermis and epidermis layers constitute a barrier for various external substances and perform a number of physiological functions, such as thermoregulation or fluid homeostasis [49]. Conventional two-dimensional cultures of keratinocytes and fibroblasts are not able to imitate the structure of the skin, because within this organ, there are also hair follicles, melanocytes, Merkel cells, blood vessels and nerve fibers. For this reason, scientists are focusing on creating microfluidic systems that are capable of simulating critical and common skin diseases [50]. In recent years, many microfluidic studies have been carried out, which can be successfully used to test the effectiveness of drugs and cosmetic products. Below are just a few examples of recent skin models. 

Jeon et al. [51] present a skin-on-a-chip model using keratinocytes and fibroblasts to study the skin response to various concentrations of sorafenib—an anti-cancer agent. The authors were able to observe side effects of this drug, which analogically occur in real patients. The formation of vascular endothelium with layers of dermis and epidermis has also been studied by this team [52]. Scientists managed to preserve the phenotypes of skin and endothelial cells during the experiment. Tissue immune response was also observed when exposed to UV radiation. Improving systems to reflect both static and dynamic conditions may be the key to further advancement. Lee and Sung [53] showed how different breeding conditions influence the course of the experiment. They demonstrated a significant impact of collagen scaffold thickness and microchannel size on cell survival. The ability to deposit keratinocytes in the system depending on the change of fluid dynamics was also observed. Research to date provides a lot of valuable information that can be used to develop new microfluidic platforms to study physiology and to develop new drugs.

### 3.8. Lymph Node-on-a-Chip

The immune system involves many complex processes with different types of cells located in central and peripheral lymphoid organs. In vitro studies using tissue and organoid models to mimic human immunity are quite modest. These traditional methods of cell culture are very limited due to the lack of extracellular matrix, haptotactic gradients, shear stress, and other hemodynamic forces influencing intercellular interactions [54]. 

In order to understand the mechanisms responsible for immune processes, Sardi, Lubitz, and Giese [55] developed an artificial lymph node model (HuALN) as a tool for testing biopharmaceuticals and vaccines to assess immunomodulation, immunogenicity, and immunotoxicity. This model uses peripheral blood mononuclear cells (PBMCs) in a perfusion bioreactor system. Thanks to this system, the expression of adhesion molecules, which are typical of stromal cells, was induced, and the most appropriate source of mesenchymal stem cells (MSC) was selected. Differentiated cells have also been shown to interact with immune cells, regulating antigen-stimulated cytokine production. The human lymph node was also developed by Shanti et al. [56], where different types of immune cells were used. This system was produced thanks to CNC (Computer Numerical Control) machining with an associated clamping system. The advantage of this system was the possibility of real-time microscopic imaging. In this study, the extracellular matrix composition was similar to the native one, and the morphology, porosity, stiffness, and permeability were similar. The division of immune cells into structural domains, replication of the lymph fluid flow pattern, and intercellular interactions across ventricular boundaries were also observed. Intercellular dynamics and lymph node physiology were investigated in Rosa et al. [54], who showed the random migration of antigen-specific T cells depending on different shear stresses [54]. According to the authors, this model enables the study of intercellular signaling of immunological synapses, which will contribute to the development of immunotherapy.

### 3.9. Body-on-a-Chip

The results of previously described studies involving microfluidic systems render information on physiology and tissue responses to a number of mechanical and chemical factors. Nonetheless, a somewhat more holistic approach is required to perform a thorough analysis of compound efficacy that could replace current solutions based on preclinical animal models. Devices consisting of biomimetic organs modules connected in one circuit by means of a perfusion system are promising solutions for studying the interaction of organs and the impact of a given active substance on non-target organs [57].

An example of a multi-organ microfluidic system is described in Oleaga et al. [58], which is a model consisting of the heart, liver, skeletal muscles, and the nervous system. This platform was capable of maintaining the culture for 28 days in gravity-induced flow, which is the minimum time for toxicological tests in standard animal models. In another study, Satoh et al. [59] created a multi-system integrated circuit placed on a platform, in which the culture medium was circulated by means of pneumatic sequential pressure. This type of flow system allowed an even distribution of components that were marked with a fluorescent dye. The device enabled the evaluation of the effectiveness of three anti-cancer drugs in four-organ systems: liver, intestine, cancer, and connective tissue. One of the results from this research that is worth mentioning is demonstrating the indirect action of the capecitabine prodrug, whose metabolite in the liver system inhibited the proliferation of cancer cells. A different but still very thought-provoking "body-on-a-chip" system is the platform designed by Miller and Shuler [60], based on the physiological pharmacokinetic–pharmacodynamic model (PBPK-PD), in which a channel connection of 13 organs was used to study interactions and responses between different cell lines. The platform consisted of a barrier chamber corresponding to the lungs, skin, and digestive tract. This layer was directly exposed to external factors and separated by a membrane from cell lines posing as kidneys, heart, liver, spleen, pancreas, bone marrow, brain, muscles, and adipose tissue. During the experiment, the researchers managed to maintain high cell viability and make a series of measurements of the physiological functions of cells. The examples cited above illustrate how integrated chip technology can be used for the further development of tissue engineering. It is very clear that building new and effective body-on-a-chip platforms requires the involvement of both engineering and medical fields [61].

### 3.10. Limitations and Perspectives of Organ-on-a-Chip Platforms

Despite the high level of sophistication of integrated organs-on-a-chip, this technology still faces many challenges. One of the problems limiting OOC as a faithful resemblance of the in vivo environment is obtaining a fully functional vascular system. Currently available solutions such as microflows enable only simple systems aimed at delivering substances to 3D hydrogel constructs containing tumor spheroids or cells representing the appropriate organ. The use of organ-specific endothelial cells and building an efficient network of vessels from these endothelial cells would allow for a better understanding of organ physiology and conducting long-term toxicological tests, since a high level of oxygen supply and other substances is necessary for proper development of the substance [46]. The material from which microfluidic systems are made also causes a significant problem. Currently, the most commonly used material is PDMS (polydimethylsiloxane), which, despite many advantages, such as transparency, gas permeability or compatibility, has also many limitations. The poor resistance of PDMS to organic solvents makes this material show a tendency to absorb small hydrophobic molecules, including drugs and fluorescent dyes. Transport, mechanical, and structural properties are also questionable as analogous to natural tissue–tissue membranes. Obtaining the right hydrogel that is able to provide cells with appropriate spatial architecture, biochemical composition, as well as mechanical properties similar to those in the extracellular matrix is a common challenge in the field of biomedical engineering. However, in case of organs-on-a-chip platforms, chemically modified "intelligent hydrogels" are additionally developed to serve as biosensors to track changes such as pH, topography, electrical conductivity, and metabolic processes and cellular responses [62]. Scalability is another important issue in the implementation of microfluidic systems for preclinical studies. Mechatronic, mesoscale, and computer mathematical modeling methods are necessary to transfer the scale from in vivo to in vitro models and to preserve the appropriate conditions in an integrated modular system [63]. Achievements in many areas have contributed to the creation of the OOC system, which, thanks to continuous improvements, becomes a real alternative to in vivo testing. However, despite the development of this technology, it is still not enough to replace researching on an animal model. The main limitations are the lack of a functional vascular system enabling the exchange of nutrients and gases and the inability to restore the structure of native tissue. The solution to these restrictions is the use of 3D bioprinting technology. 

## 4. 3D Bioprinting

The emergence of 3D bioprinting has revolutionized biomedical technology, introducing the possibility of maintaining 3D cell cultures. Bioprinting is becoming increasingly popular in the field of regenerative medicine as well as in the pharmaceutical industry. Currently, this technology is the basis for the development and improvement of bionic models of tissues, organs, organoids, and organs on the chip [64]. 

The main goal of 3D bioprinting is to restore the natural microenvironment of a given type of cells—the extracellular matrix. It is a 3D structure that on the one hand provides a mechanical scaffold to support cells, and on the other hand is a complex biochemical environment ensuring cells’ proper functioning and well-being [65,66,67]. Hydrogels are commonly used to create 3D cellular scaffolds. They are built from hydrophilic polymer networks that show a cell-friendly environment. The occurrence of cross-covalent bonds or bonds resulting from electrostatic interactions determine the mechanical properties of the obtained crosslinked hydrogels.

Hydrogels used for bioprinting are present in liquid form as polymer solutions. After obtaining a structure or a monolayer, appropriate crosslinking agents are used. For this reason, it is critical to examine the fluid properties before crosslinking and to research the mechanical properties of the object after crosslinking. Important criteria to take into account while assessing bionic properties are thinning, shear modulus, shear recovery and stress relaxation [68]. 

Basic materials used in the bioprinting process include collagen, alginate, hyaluronic acid, gelatin, cellulose, and chitosan. Hydrogels obtained from organ decellularization are also used quite often [69]. Not to mention, more and more research teams focus on obtaining extracellular matrix from native tissues through decellularization (dECM) [65,66,67]. 

Currently, several decellularization strategies are being developed using different physio-chemical phenomena such as cyclic freezing and thawing [70], high hydrostatic pressure [71], exposure to supercritical gases [72], and (the most common of them all) the use of surfactants [73], [74]. Regardless of the decellularization technique used, it is extremely important to achieve the right quality of a final product. This applies in particular to the absence of detergent residues (i.e., potent toxic residual DNA content must be less than 50 ng/mg of dry weight of the dECM) and to the removal of body fat present in the native organ [75]. In addition, the protein content of the ECM should be evaluated with attention to structural proteins: collagen, fibronectin and laminin, glycosaminoglycans (GAG), and growth factors [76]. 

Rebuilding a native tissue (including entire organs) using 3D bioprinting requires great precision in the spatial distribution of cells, growth factors, drugs, or other biologically active elements. Viable cells are constantly exposed to mechanical stimulation from both the extracellular matrix and adjacent cells [77]. Cell–cell and cell–ECM interactions affect, among others, cell metabolism, protein synthesis, the breakdown of cytoskeletal proteins, cell survival and, consequently, cell mechanics, i.e., migration, cell expansion, and contraction [78]. That is why it is extremely important to reproduce the structure of the original cell environment as accurately as possible.

**Figure 4:**
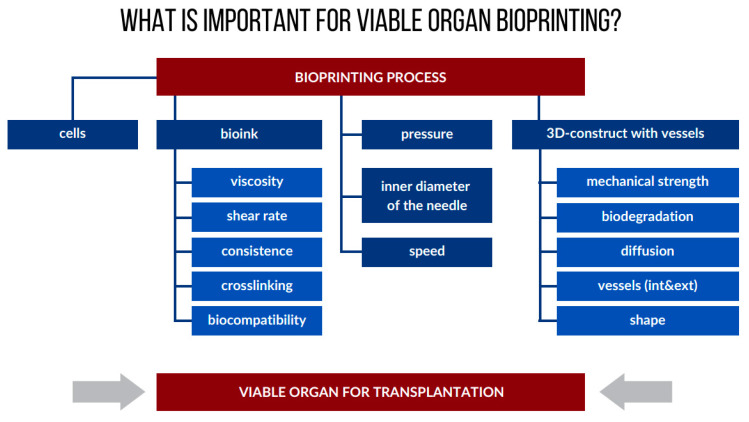
What is important for viable organ bioprinting? Parameters are based on the most commonly used 3D bioprinting technique—extrusion bioprinting.

From a technical point of view, bioprinting enables a controlled and automated production of live biological models [79]. However, depending on the carrier used, i.e., the biomaterials in which the cells are embedded, this process requires a thorough optimization of individual stages. The most important aspects of this include (Figure 4):

- preparation of a bioink for biological printing (e.g., with appropriate printability—viscosity, consistency, shear rate) that has a composition mimicking the native cell environment;
- optimization of the conditions of the bioprinting process, e.g., printing speed, pressure applied to the bioinks in the extrusion method, inner diameter of the needle;
- assessment of the final bioconstruct, e.g., mechanical strength, biodegradation, diffusion;
- monitoring the condition of cells subjected to the bioprinting process, e.g., viability, proliferation, functionality.

## 5. Methods of 3D Bioprinting

**Figure 5:**
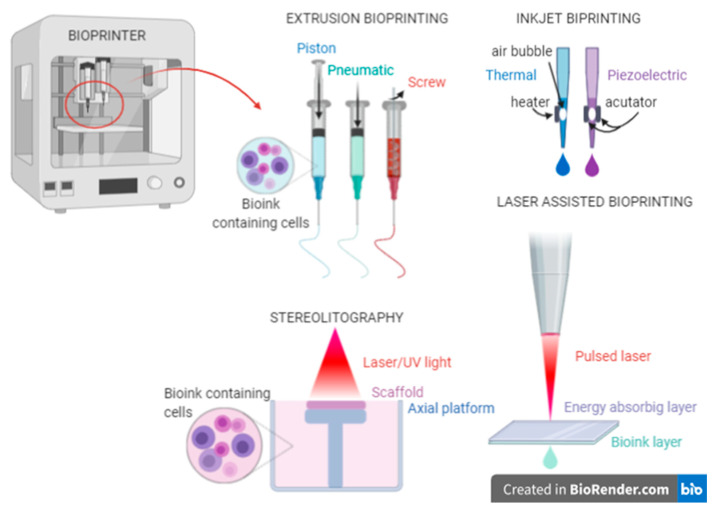
Commonly used 3D bioprinting methods.

There are several 3D bioprinting methods that may be used depending on the properties of the exact bioink. A summary of these methods is presented in Figure 5. We also prepared short comparison of these methods and collect data in Table 1. 

### 5.1. Extrusion-Based Method

There are several 3D bioprinting methods comprising cell-based bioinks. One of the most commonly used techniques in bioprinting is the extrusion method. It relies on extruding biomaterials from cartridges by mechanical forces or pneumatic pressure onto the platform in a continuous manner, thereby obtaining unbroken cylindrical lines. The diameter of the extruded fiber results directly from the size of the nozzle used for a particular type of material. Temperature, pressure, piston, and rotational speed are controlled by a computer, and the working area is based on the XYZ axis. The extrusion method allows for the use of bioinks with high cell density and for embedding them in desired places throughout the planned construct. The controlled heterogeneity of the bioprinted structure is particularly important in building larger tissues or entire artificial organs and permits better reflection of physiological processes [102]. Another advantage of this method is the possibility of using a wide range of hydrogel polymers and prepolymers with viscosities in the range of 30–6 × 10^7^ mPa/s and relatively inexpensive bioprinting accessories [103].

Commercially available bioprinters are not limited to one method. Modern bioprinting solutions combine several modules such as extrusion, electrospinning, UV crosslinking and temperature control inside the print head and on the printing surface [102]. One of the limitations of this method is the direct exposure of cells to mechanical stress. Shear stress caused by high pressure in the nozzle causes a decrease in cell viability of up to 40–85%. A larger nozzle size and the use of lower pressure values reduce the expenditure but cause a significant loss of resolution and printing speed. The impact of UV radiation on crosslinked hydrogels is also significant. Therefore, due to these limitations, it is necessary to assess the viability of the cells and their functionality after the printing process. Wszola et al., as part of the ongoing bionic pancreas project, showed that crosslinking with 405 nm is better for pancreatic islets than crosslinking with 365 nm UV light. Research is currently focused mainly on developing optimal bioinks and introducing control systems [104,105].

### 5.2. Inkjet-Based Method

Inkjet bioprinting is another method used that involves ejecting individual droplets with a controlled size from the nozzle. This happens by means of piezoelectric or thermal force mechanisms. The advantages of this technique are maintaining high cell viability (80–90%) and high printing speed. Obtaining high cell density in this method is impossible, since it is correlated with increasing the viscosity of the biomaterial, which leads to clogging the nozzle. Therefore, materials used in this technique cannot exceed a viscosity threshold of 10 mPa/s. Due to the inability to construct solid three-dimensional structures, this technique is used less often [85,106].

### 5.3. Laser-Assisted-Based Method

Laser-assisted bioprinting is a less common technique used in tissue engineering. The laser-induced system consists of a pulsating laser beam, a focusing system, and a so-called ‘ribbon’. The ribbon structure is made of two layers: the upper—energy absorbing, and the lower—a suspended bioink. The laser pulse induces the evaporation of the upper layer, and a bubble is formed at the interface. This leads to the bioink drop being transferred to the surface and then crosslinked. This method aims at obtaining highly complex three-dimensional geometries [107]. Nevertheless, because of the complicated handling of the laser system and expensive parts, this technique is less commonly used.

### 5.4. Stereolitography-Based Method

Of all the techniques used in bioprinting, stereolithography (SLA) has become one of the most regularly used in tissue engineering. This method is based on a tank filled with a solution of photo-curable polymer, a laser, controlled movements in the XY axis, and a movable platform in the Z axis. The first step of this technique is hardening the biomaterial of a single layer with a beam of light reflected in micro-mirrors. Then, the platform lowers the position to get to the next layer, resulting in a 3D structure.

In comparison with other methods, stereolithography is characterized by high resolution (<100 μm), fast bioprinting process, high cell viability (>85%), and the possibility of obtaining large 3D objects with precise and controlled surface properties. Biomaterials used in this technique must have in their structure appropriate groups that are capable of photopolymerization through UV radiation (range from 365 to 405 nm) in the presence of a photoinitiator in the range from 365 to 405 nm. The most commonly used materials in this method are proteins: gelatin methacrylate (GelMA), collagen methacrylate (ColMA); and non-proteins: hyaluronic acid methacrylate (HA), polyethylene glycol diacrylate and dimethacrylate (PEGDA, PEGDMA). 

Although this technique is one of the most promising in the field of tissue engineering, it has several restrictions. It has been shown that the use of UV light can damage cell DNA. Nevertheless, new possibilities are emerging from using crosslinking with the 522 nm in the presence of eosin Y. Another problem is organizing cells in a controlled manner due to the fluidity of the biomaterial [103,108].

## 6. 3D Bioprinting of Organs-on-a-Chip

The growing demand for microfluidic models in the field of tissue engineering requires using advanced techniques that will simplify the production process of microfluidic devices, as well as the precise placement of biological material in these devices. Bioprinters based on technologies such as stereolithography (SLA), Fused Deposition Method (FDM), Two-photon polymerization (2PP), digital micro-projection projection bioprinting (DMD-PP), and extrusion bioprinting are commonly used for such devices. The materials employed in bioprinting are mainly photosetting resins and thermoplastic synthetic polymers. Three-dimensional bioprinting has also found great use, allowing not only for the precise distribution of biological material, but also for obtaining high-quality channels, even in a 3D structure. Microchannels obtained using bioprinters can range from 100 to 300 µm in diameter with great accuracy [24]. For the precise deposition of cells, media, or tissue scaffolds, the bioprinters described above in this article are used.

Bioprinting is a revolutionary technology that can be used to create scaffolds for tissue culture. Recently, there have been many scientific reports in the literature on the subject of microfluidic organs on a chip. They are multifunctional, highly useful platforms with wide application mainly in drug screening and pathological tests. The organ models created in this system are used for research to summarize the structural, microenvironmental, and physiological functions of human organs. Lately, due to a number of advantages of bioprinting technology, it was decided to use it to produce organ models on a chip employing many materials and cell types simultaneously with very good spatial resolution and reproducibility. The combination of bioprinting techniques with the concept of the organ-on-a-chip system enables the creation of a biomimetic microenvironment with heterogeneous 3D structures. Functional vascularized tissue structure can be printed directly, allowing fluid to flow. Modern biomedical engineering focuses particularly on the integration of microfluidic and bioprinting technologies to generate more complex tissues or organs and advanced in vitro flow systems.

Table 2 summarizes examples of a combination of bioprinting and microfluidics to generate complex tissue/organ models.

Despite the widespread interest in combining microfluidic systems and bioprinting into biomedical solutions to create more advanced tissue structures, a number of options require further improvement. Several problems remain to be solved. The most common problem is choosing a printing method with consideration to printing with biological material. Extrusion printing is used for this purpose, and it is gaining popularity due to its low cost and mild printing conditions [120]. However, because of the limited resolution and surface roughness, it is not used as a microflow platform with complex heterogeneous 3D structures. Another method—SLA laser printing—can achieve high resolution, but the choice of biomaterials is limited, and cells cannot be printed together with the scaffolding without damaging the cells [121]. Observing the growing interest in combining the microfluidic system and bioprinting, there is a trend of printing a tissue model with a microfluidic chip in one production stage [110]. One-step production process enables more efficient and automated process flow. For this reason, bioprinters enabling the printing of both the flow system and tissue at once are used. In addition, it seems important to improve the system with a sensor system to monitor the behavior of cells and their microenvironment, which is another challenge for scientists and is crucial for clinical applications.

Another challenge is choosing the right material. Several commonly used resin materials in the precision printing process are cytotoxic and have limited optical transparency. In addition, unlike the commonly used microfluidic systems based on PDMS, most 3D-printed biochips cannot be autoclaved [122]. Nevertheless, with the development of the processes of bioprinting and bioinks, we expect that highly efficient, automated, and integrated platforms for printing organs on microchips can be widely used to create functional tissues.

## 7. The First Bionic Organs

The main goal of 3D bioprinting strategies is to design biological tissue constructs that can be implanted in the body. Currently used 3D bioprinting technologies allow the production of spatial structures of any shape with many types of cells using a variety of biomaterials, resulting in complicated constructs that can replace human tissues or organs. Bioprinting technologies offer many opportunities in the field of biomedical engineering, from simple shape-based tissues such as bone, cartilage, skin, and cornea, through highly organized tissues such as skeletal muscle, heart muscle, and nerve tissues to complex intermediate tissues such as bone–cartilage tissues and finally, to complete organs with vessels and functional internal structures such as the liver, kidney and heart. The efforts to deliver clinically bioengineered tissues or organs are steadily advancing; in the long run, there is hope that tissue engineering will have a significant impact on improving patients’ lives.

**Figure 6:**
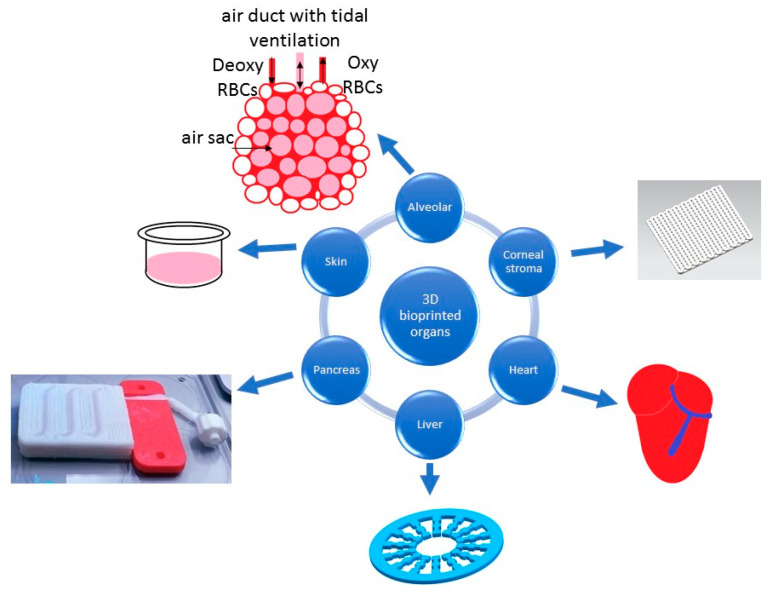
Diagram showing present uses of 3D bioprinted organs. All figures within this figure were prepared on the basis of original papers (alveolar [125], corneal stroma [126], heart [127], liver [128], pancreas (private photo), and skin [129]).

Despite the many difficulties that the field of biomedical engineering brings with it, 3D bioprinting technology seems to be the right solution to revolutionize the medical and scientific world in the coming years. Currently, there is a lot of research on the use of 3D bioprinting to create such tissues as blood vessel, heart, bone, liver and skin. It is worth mentioning that at this stage, all models are applicable only to laboratory tests, although recently, there have been reports of the production of functional artificial heart, lung, and pancreas [123,124]. Examples of 3D bioprinted organ models are presented in the Figure 6.

### 7.1. Liver Model

Lee at el. [130] attempted to develop alternative in vitro liver models for transplants and drug screening. Despite many studies, a full mapping of organ complexity has not been obtained. The group has developed a dECM-based biomaterial optimized for the need of liver tissue engineering in 3D printing and has determined the printing parameters, using it to map the 3D liver model. As part of the study, stem cell differentiation and HepG2 cell function in liver dECM bioinks were also assessed compared to commercial collagen bioinks. dECM bioink induces stem cell differentiation and improves HepG2 cell function. The results show that the proposed bioink dECM from the liver is a promising candidate for liver tissue bioink engineering based on 3D cell printing [130].

Mao et al. [128] developed an innovative model of hepatic microtissue, in which a significant body surface area was achieved in order to improve or restore the functionality of the organ. As part of the work, a specific bioink for liver tissue was developed by combining photosetting methacrylated gelatin (GelMA) with extracellular matrix (dECM) and hepatocytes (hiHep). The mechanical properties, swelling, and compatibility of GelMA/dECM bioinks have been thoroughly characterized before 3D printing. Based on the research, it was found that dECM improves both the printability and viability of cells and the functionality of hiHep cells that spread and perform their specific functions more efficiently (albumin secretion and urea). The result of this study is the development of material suitable for printing liver micro tissues that can be used in liver tissue engineering to restore liver function [128]. 

### 7.2. Skin Model 

A Spanish group of researchers has developed a method for producing double-layered human skin by 3D bioprinting using bioinks containing human plasma and primary human fibroblasts, and keratinocytes from skin biopsies. Using this method, 100 cm^2^ of bioprinted skin was obtained in less than 35 minutes. In addition, the bioprinted structure was analyzed using histological and immunohistochemical methods, in both in vitro 3D cultures and after transplantation in immunodeficient mice. Bioprinted skin has been shown to be similar to normal human skin and cannot be distinguished from bilayer cutaneous and epidermal counterparts. Therefore, it can be successfully used in the clinics [131]. 

### 7.3. Corneal Stroma Model

Recently, there have been reports on cells laden 3D bioprinting of the corneal stroma. In this study, a three-dimensional equivalent of the corneal stroma was designed to replace native tissue. Satisfactional tissue mapping was obtained by using optimal printing conditions. Bioprinted GelMA constructs were stable in phosphate-buffered saline (PBS) for three weeks of incubation (weight loss by 8%). The cell viability test showed 98% viable cells on day 21 of the study, indicating that the bioprinting conditions were appropriate for the treatment of keratocytes. The values of mechanical parameters within cells of 3D bioprints were increased twice during the incubation period and approached the properties of the native cornea. The expression of type I and V collagens and proteoglycan (decorin) in keratocytes indicates the maintenance of the phenotype in hydrogel prints. The transparency of the material used was high—over 80% (at 700 nm) for three weeks of culture and was comparable to the native cornea (85%) at the same wavelength. As a result, hydrogel constructs with keratocytes with appropriate biological and physical properties mimicking the native features of the corneal stroma with excellent transparency, adequate mechanical strength, and high cell viability were bioprinted [126]. 

### 7.4. Alveolar Model

Grigoryan et al. [125] developed functional entangled multi-vessel networks with high efficiency of intercellular oxygen transport between the strands and the surrounding environment, which consequently contributed to further research on the development of a biology-inspired alveolar model. The group bioprinted a model with a porous topology using the synthetic polyethylene glycol diacrylate (PEGDA) polymer. Cyclic ventilation of the connected airways with humidified oxygen gas (10 kPa, 0.5 Hz) has led to a noticeable dilatation and visible change in the curvature of the concave airways [125].

### 7.5. Heart Model

Research group Noor et al. [127] bioprinted a human heart with a natural morphology based on a hydrogel containing two types of cells, which formed the material for bioprinting the parenchymal tissue of the heart and blood vessels. Work continues to improve the vessel network through modeling oxygen transport in the system. In addition, the bioprinted construct is tested for structure and function in vitro and then morphologically evaluated after transplantation [127].

### 7.6. Pancreas Model

In 2019, the research team of Wszola developed a functional model of the pancreas with full vasculature. Scientists created two different hydrogels, both based on dECM. For the preparation of those bioinks, the dECM was obtained from a pig pancreas in the process of decellularization. The final product was characterized by the lack of any detergent, low content of lipids, and high content of collagen in comparison to the native tissue. Performed experiments proved that those properties have a positive impact on islet viability and functionality. The former bioink is intended for bioprinting the vasculature system of bionic pancreas. That is the reason why is has to be much more liquidous than the latter one, which is responsible for the creation of the whole body of bionic pancreas. The physicochemical properties of prepared bioinks have been thoroughly characterized by the rheology, compression, degradation, and printability, such as fiber continuity and smoothness. Moreover, the scientific team defined the most suitable ratio of islets to bioink and the best parameters of the 3D bioprinting process such as time of UV crosslinking and pressure used to extrude the bioink’s fiber. The viability of islets inside the obtained bionic pancreas were examined by the live/dead staining with the use of AO/Pi. Their functionality was determined by the glucose stimulated insulin secretion assay (GSIS assay), and the patency of vascular channels was confirmed by the magnetic resonance imaging with phase contrast. Presently, the publications are in preparation [75,104,124,132].

## 8. The Future of 3D Bioprinting in the Creation of Tissue Systems

Three-dimensional bioprinting technology in the field of tissue engineering provides a lot of possibilities for mass production of biocompatible and more importantly, personalized constructs. Its dynamic development is particularly noticeable in the fields of biotechnology and medicine. One of the major milestones in 3D bioprinting is the production and the most accurate reproduction of a cellular microenvironment in an autonomously ordered way for tissue engineering and regenerative medicine. Pharmaceutical and cosmetics companies also have hope for this new technology, apart from scientific and research centers, which see great potential in testing new substances precisely on 3D biosensitive tissue models.

The key research problems encountered by most scientific teams at the moment is the lack of optimized bioinks suitable for bioprinting tissue models directly with cells, so that after the whole process, cell viability is at least 80%. Another limitation is the bioprinting of the vascular system, which will ensure the adequate delivery of nutrients to cells used throughout the process. At this stage, successful conceptual studies of the bioprinting process have been carried out and mainly relatively simple miniature models that do not require vascularization have been produced. However, when the plan is to bioprint more complicated models (larger than 200 μm), bioprinting the vascular system becomes an indispensable component that will allow the diffusion of oxygen and nutrients within the entire model, and thus the functionality of the cells and ultimately the whole model is disturbed. One should not forget to optimize such parameters as the pressure and speed of printing, as well as the method of crosslinking bionic structures [123]. For pressures used throughout the process, these include the type of cells and whether organoids or microcircuits (e.g., pancreatic islets) are used in the process [133,134].

Despite the intensive work on the 3D bioprinting of tissues and organs, the replacement of native organs is a distant future. However, this does not mean that the effects of work on bionic models will take a long time. In the first place, the creation of fully functional tissue models can significantly revolutionize the pharmaceutical market [123].

It is worth noting that currently, in order to introduce one candidate for a drug to the clinic, it takes even 12–15 years of time-consuming and expensive research, and yet the risk of drug ineffectiveness or toxicity at various stages of work is almost 50% [135]. The use of bionic models with a full vascular system can significantly reduce these factors. More importantly, it can also reduce the number of animals used for scientific and preclinical research. Presently, throughout the entire process, millions of animals are used annually for medical research [136].

It is estimated that more than 100 million animals are killed every year worldwide during scientific, cosmetic, biomedical, and pharmaceutical research studies [137]. With this respect, concerns about reduction in animal usage during preclinical studies as well as improvement in animal welfare are ethically justifiable and novel solutions are awaited. Application of in vitro and/or animal models in drug development phase aim to verify effectiveness, lack or limited toxicity, and confirm good pharmacokinetics (PK) of a drug candidate. Additionally, preclinical studies are about to determine the initial dose for a potential drug required in the first phase of clinical trials and to gather as much information about the novel compound as possible. Pharma companies spend a lot of money on clinical trials and drug development processes, and still, there is low likelihood that the drug achieves market approval, since the vast majority of drugs in clinical trials does not reach market approval due to toxicity, lack of efficacy, poor PK, etc. [138]. It is well-known that it may result from the utilization of unreliable 2D in vitro cell cultures or animal models with limited predictive value, since there are major discrepancies between animal and human studies that lead to confounding results. Testing drugs on animals has always been controversial, since animal models of disease has disadvantages including high costs, limited reliability, feasibility and/or availability. Finally, drug development is time-consuming; therefore, any solution that may accelerate the selection of best drug candidates for further clinical development is strongly needed [139]. 

It is well-known that animals share many emotional and cognitive characteristics with humans. Due to ethical concerns, it is important to provide the replacement for animals’ studies by the application of novel scientific approaches. The 3Rs rule (Replacement, Reduction, and Refinement) recommends that animal testing shall be reduced or replaced by other available methods. Moreover, an animal’s pain and discomfort shall be minimized in order to positively impact their welfare. With this respect, the incorporation of 3D bioprinting into preclinical drug development may become an important alternative for animal testing, providing a quick and reliable answer on drug characteristics, since many compounds can be screened at a time, giving robust answers. Three-dimensional bioprinted cells, tissues, or organs may grow in vitro to create living structures. It is assumed that such structures replicate the behavior and functions of the human body, since they are aimed to fabricate 3D structures that could display the basic characteristic and physiology found in natural organisms such as cell–cell interactions, cellular microenvironments, and the complexity of cellular pathways. Additionally, in personalized therapies, it is possible to generate platforms that test each patient’s response to drug therapies in vitro. It is more ethical, since it may provide more reliable data in terms of drug efficacy, potency and tissue toxicity. Finally, the 3D bioprinting of human tissues may reduce the predictability gap between in vitro and in vivo assays and clinical trial outcomes. Fabricating functional tissues, organs, organoids, spheroids, organ-on-chip, etc., may become an important tool for research and science development [140,141,142,143]. 

As an example, the fabrication of human skin using 3D bioprinting technology is the approach that may lead to significant advancements in the field of autoimmune diseases, tumors of the skin, and many others, giving a powerful options for translation of preclinical data into more realistic modeling, while reducing the reliance on in vivo animal models. As an example, the preliminary data on the biofabrication of 3D bioprinted skin model was presented in terms of the reproduction of key morphological and biological characteristics of in vivo human skin, demonstrating the effective control of cell location, density, and number of layers [98]. Moreover, novel platforms for drug candidates of skin diseases must be validated, since preclinical in vivo models cannot completely predict their usability in clinical settings (i.e., pharmacokinetics parameters) because skin structure and organization vary between mammalians [144]. With this respect, there is a need for the replacement of current in vivo procedures with in vitro techniques that will closely reflect the physiological conditions in the human body in order to provide non-animal testing on therapeutics using 3D cell culture platforms. 

Reliable drug testing systems are urgently needed, since obtaining an accurate drug response that reflects realistic drug efficacy/toxicity in vivo (and further in the clinic) is a major goal for drug development studies. To investigate tumors’ biology and drug response, one of the studies demonstrates a biofabrication method that combines a spheroids-forming technique with 3D bioprinting methods. The results suggest that this system is very accurate not only for in vitro drug testing and research studies, but it also provides a suitable platform for the further reduction of animal study engagement during preclinical settings [145]. Moreover, 3D bioprinting may contribute to the creation of more reliable and repeatable animal models of disease. With this respect, the number of animals will also be reduced, because this model will be more accurate; thus, no additional confirmatory animal models will be further required. In one study, the biofabricated novel device with the use of a 3D bioprinter was used to create a novel animal model of TNBS (2,4,6-trinitrobenzene sulfonic acid)-induced colitis. This model may become a useful tool to study future therapies for inflammatory bowel disease (IBD) [146]. Additionally, 3D models may be used in education, as an alternative for demonstrations or vivisections in terms of the visualization of internal structures and disease pathology, as well as the comprehension of cellular processes or organ activity. With this respect, the technology of 3D bioprinting is a valuable tool for constructing human anatomical models for preoperative planning and education purposes. One study demonstrates the creation of an endovascular stimulator with patient-specific vascular anatomy in order to realistically mimic endovascular procedures. This approach not only helps in increasing training abilities or enhancing the rate of further development of new therapies, but it also reduces the use of animals during the testing process of new devices in this field [147]. It is assumed that the commercialization of functional and vascularized tissue models produced by 3D bioprinting technology may reduce the amount of animals used for scientific purposes by 23–55% [148,149].

However, improvement of this technology is still required. One of the challenges for the extrusion 3D bioprinting method is the ability to achieve high precision, e.g., to reproduce capillaries with diameters up to 20 μm. In this respect, stereolithographic bioprinting methods such as DLP (digital light processing) undoubtedly win over extrusion or inkjet methods. Nevertheless, due to the complexity of tissue structure, differences in the size, stiffness, and type of cells, standard methods of extrusion are more versatile, mainly because of the possibility of applying layers of materials with specially dedicated purposes [150]. However, technological advances in bioprinting and the development of biomaterials reproducing a realistic environment for cells are not able to fully meet the needs of tissue engineering. Innovative devices such as bioreactors or microfluidic systems capable of maintaining appropriate conditions for long-term tissue culture are also needed [105].

## 9. Summary

Nowadays, 3D bioprinting and organs-on-a-chip are great alternatives for commonly used techniques. Bioprinting technology allows constructing live and functional 3D constructs, which may be a replacement for imperfect animal models used in cosmetics and pharmaceutical industries. Moreover, bioprinted constructs are much more controllable and repeatable than presently used animal models. They give much more reliable data and may contribute to a decrease or even fully rule out animals in various tests. It is worth mentioning that the development of modern chemical and material engineering has contributed to the development of bioprinting technology and the organ-on-a chip system. The latter is an excellent tool for studying molecular mechanisms of disease development and the pharmacokinetics of various types of cells within one or more organs. OOC technology, due to controlled conditions and the ability to monitor intercellular interactions, is a particularly promising solution for the rapid, initial evaluation of drug toxicity, which can significantly reduce the number of preclinical studies. However, OOC is not able to fully map organ function, chronic disease physiology, and immune responses. Merging several systems at the same time is also a problem. It must be kept in mind that 3D bioprinting has also many restrictions. The main concern is bioink, which must represent the proper printability and biological and physical features (consistency, composition mimicking native tissue, and viscosity). According to the World Health Organization (WHO) Global Observatory on Donation and Transplantation, there are over 130,000 solid organ transplantations performed annually worldwide. However, it is estimated that this number covers only about 10% of actual needs. With this respect, solid organ shortage may be overcome with the use of 3D bioprinting techniques, where such a 3D bioprinted vascularized organ composed of live cell embedded in the extracellular matrix may be transplanted into a patient. This approach is an excellent alternative for current therapies, since it is more patient-specific, less expensive, and faster in term of the patients waiting list for donation. Interestingly, the cells in a 3D bioprinted organ may originate from autogenic transplantation (e.g., pancreatic islet cells) or they may be stem cells, which may be further transformed into organ-specific cells. Such an approach allows to escape from an immune attack toward a transplanted 3D bioprinted organ, finally resulting in the minimization of organ rejections. There are still some challenges, including proper organ vascularization or stem cell usage; however, in the near future, significant progress in the field of organ transplantation is expected to be achieved by the use of 3D bioprinted techniques and methodologies.

To conclude, it is believed that both 3D bioprinting and OOC will have a significant impact on healthcare around the world. Developments in the field of 3D bioprinting and chip systems provide novel platforms to follow trends toward non-animal testing and fulfill unmet medical needs such as regeneration medicine, transplantation, etc. These technologies are of great potential to eliminate testing on animals and provide patient specific drug testing. Other benefits include enhancing experimentation capabilities and savings in funding. The 3D cell culture obtained in the bioprinting process mimics the spatial organization of cells in a living organism, while OOC allows monitoring intercellular interactions; thus, testing the activity of drug candidates is more predictive and valuable in these assay systems. Therefore, 3D bioprinting and OOC shall become an important tool in preclinical studies as well as research activities. The bioprinting of organs that resemble human nature can limit the animal usage in research and pharma studies. The utilization of 3D bioprinting and OOC in drug candidate screening allows for the better selection of potential therapeutics for further development in clinical studies, hence reducing the failure rate and providing an alternative for animal models.

## Figures and Tables

**Table 1:** Comparison of commonly used 3D bioprinting methods. ECM: extracellular matrix.

Feature	Microextrusion	Inkjet	Laser	Stereolithography	Ref.
**Dosing method**	fiber	drop	drop	tank	[80,81]
**Density of cells**	high + spheroids	10^6^–10^7^ cells/mL	10^6^–10^7^ cells/mL	< 10^8^ cells/mL	[82]
**Cells viability after bioprinting process**	40–95%	> 85%	> 95%	> 90%	[80,81,82,83,84,85,86]
**Maximal bioink’s viscosity**	high 6 × 10^7^ mPa/s	low 10 mPa/s	medium 300 mPa/s	medium-high 2,000 mPa/s	[80,81]
**Bioprinting speed**	low 10–50 μm/s	medium 1–10 000 drop/s	high 200–1,600 mm/s	high < 14 mm/h	[87,88] [82,83,85,86,87]
**Resolution**	100 μm–1 mm	50–300 μm	10–50 μm	< 100 μm	[82,83,85]
**Crosslinking method**	chemical, light, thermal, enzymatic, ion, shear, pH	chemical, light, thermal, enzymatic, ion	chemical, light ion	light	[82,84,87,88]
**Integrity of the final structure**	high	low	low	high	[82]
**Cost**	medium	low	high	low	[81,82,83,84,85,87]
**Examples of used bioinks**	hyaluronic acid, gelatin, alginate, collagen, fibrin	hydrogels, agar, alginate, collagen, fibrin	hydrogels, nano-hydroxyapatite	methacrylates of gelatin, hyaluronic acid, collagen; polyethylene glycol diacrylate, dimethacrylate	[82]
**Advantages**	simple technique; suitable for various biomaterials including ECM mimetic hydrogels; enables printing with high cell density material	possibility of printing with low viscosity liquids; fast print; low costs; high resolution; low shear forces during bioprinting	high resolution; distribution of biomaterial in solid or liquid phase	nozzle-free technique; high accuracy and cell viability	[83,85]
**Disadvantages**	suitable only for viscous liquids; induces high shear forces that can affect cell viability and phenotype	continuous printing is not possible; low cell density; problems with vertical structures; may cause mechanical deformation of cells	high costs; damage caused by high temperatures during the laser pulse	UV light source and near-UV blue light’s toxicity to cells; lack of printing multicells; damage to cells during photo curing	[83,85,89,90]
**Example of uses**	trachea, heart valve	skin, blood vessels, cartilage	skin	pulmonary alveolus, blood vessel, cartilage, organ-on-a-chip	[82,91,92,93,94,95,96,97,98,99,100,101]

**Table 2:** Examples of a combination of bioprinting and microfluidics. dECM: decellularization, PDMS: polydimethylsiloxan. PMMA: poly(methyl methacrylate). PCL: polycaprolactone.

Tissue Model	3D Printing Methods	Bioink for Cells/Hydrogel	Microfluidic System/Substrate Material	Ref.
Liver	Microextrusion	GelMA and gelatin	PDMS and PMMA	[109]
		Gelatin and collagen I	PCL	[110]
	Inkjet	Alginate	PDMS and glass	[111]
Tumor	Microextrusion	Alginate		[112]
		Cell suspended in media	PDMS	[113]
Bone, cartilage and muscle	Microextrusion	Gelatin, fibrinogen, HA and glycerol	PCL	[114]
Heart, blood vessel		Alginate, GelMA and irgacure 2959	PMMA and PDMS	[115]
		Alginate and GelMA	PDMS	[116]
Kidney		Fibrynogen and gelatin	PMMA	[117]
Lung		Tracheal mucosaderived dECM (tmdECM)	PCL	[118]
Urothelium, blood vessel		GelMA, alginate and tripentaerythritol	Bioprinted construct	[119]

## Abbreviations

2PP	two-photon polymerization
3R	Replacement, Reduction, and Refinement
ECM	extracellular matrix
ALD	alcoholic liver disease
BBB	blood-brain barrier
CNS	central nervous system
dECM	decellularized extracellular matrix
DLP	digital light processing
DMD-PP	digital micro-projection projection printing
DOX	doxorubicin
FDM	Fused Deposition Method
GAG	glycosaminoglycan
GELMA	gelatin methacrylate
HA	hyaluronic acid methacrylate
HepG2	human liver cancer cell line
hiHep	hepatocytes
HUVEC	Human umbilical vein endothelial cells
NCI H1437	Human cells; non-small cell lung cancer
OOC	Organ-on-a-chip
PBPK-PD	physiologically-based pharmacokinetic/pharmacodynamic
PBS	phosphate-buffered saline
PCL	polycaprolactone
PDMS	polydimethylsiloxane
PEGDA	polyethylene glycol diacrylate
PEGDMA	dimethacrylate polyethylene glycol diacrylate
PK	pharmacokinetics
PLA	polylactic acid
PMMA	polymethyl methacrylate
PTS	precisely cut tissue slices
rhHGF	Recombinant human Hepatocyte Growth Factor
SLA	stereolithography
TEER	transepithelial electrical resistance
μTAS	Micro Total Analysis Systems
